# Caveats of chimpanzee ChAdOx1 adenovirus-vectored vaccines to boost anti-SARS-CoV-2 protective immunity in mice

**DOI:** 10.1007/s00253-023-12927-0

**Published:** 2024-01-27

**Authors:** Jacquelynne Cervantes-Torres, Carlos Cabello-Gutiérrez, Dolores-Adriana Ayón-Núñez, Gloria Soldevila, Roxana Olguin-Alor, Georgina Diaz, Gonzalo Acero, René Segura-Velázquez, Leonor Huerta, Isabel Gracia-Mora, Laura Cobos, Mayra Pérez-Tapia, Juan C. Almagro, Francisco Suárez-Güemes, Raúl J. Bobes, Gladis Fragoso, Edda Sciutto, Juan Pedro Laclette

**Affiliations:** 1 School of Veterinary Medicine, Universidad Nacional Autónoma de México, Coyoacán, 04510 Mexico City, Mexico; 2 Biomedical Research Institute, Universidad Nacional Autónoma de México, Coyoacán, 04510 Mexico City, Mexico; 3 Instituto Nacional de Enfermedades Respiratorias “Ismael Cosío Villegas”, Calzada de Tlalpan 4502, Belisario Domínguez Secc. 16, Tlalpan, 14080 Mexico City, CDMX Mexico; 4 Laboratorio Nacional de Citometría de Flujo, Instituto de Investigaciones Biomédicas, Universidad Nacional Autónoma de México, Coyoacán, 04510 Mexico City, Mexico; 5 Unidad de Experimentación Preclínica, Facultad de Química, Universidad Nacional Autónoma de México, Coyoacán, 04510 Mexico City, Mexico; 6 Unidad de Desarrollo e Investigación en Bioterapeúticos (UDIBI), Escuela Nacional de Ciencias Biológicas, Instituto Politécnico Nacional, 11340 Mexico City, Mexico

**Keywords:** COVID-19, Receptor-binding domain (RBD), Neutralizing antibodies, Cellular immunity

## Abstract

**Abstract:**

Several COVID-19 vaccines use adenovirus vectors to deliver the SARS-CoV-2 spike (S) protein. Immunization with these vaccines promotes immunity against the S protein, but against also the adenovirus itself. This could interfere with the entry of the vaccine into the cell, reducing its efficacy. Herein, we evaluate the efficiency of an adenovirus-vectored vaccine (chimpanzee ChAdOx1 adenovirus, AZD1222) in boosting the specific immunity compared to that induced by a recombinant receptor-binding domain (RBD)-based vaccine without viral vector. Mice immunized with the AZD1222 human vaccine were given a booster 6 months later, with either the homologous vaccine or a recombinant vaccine based on RBD of the delta variant, which was prevalent at the start of this study. A significant increase in anti-RBD antibody levels was observed in rRBD-boosted mice (31–61%) compared to those receiving two doses of AZD1222 (0%). Significantly higher rates of PepMix™- or RBD-elicited proliferation were also observed in IFNγ-producing CD4 and CD8 cells from mice boosted with one or two doses of RBD, respectively. The lower efficiency of the ChAdOx1-S vaccine in boosting specific immunity could be the result of a pre-existing anti-vector immunity, induced by increased levels of anti-adenovirus antibodies found both in mice and humans. Taken together, these results point to the importance of avoiding the recurrent use of the same adenovirus vector in individuals with immunity and memory against them. It also illustrates the disadvantages of ChAdOx1 adenovirus-vectored vaccine with respect to recombinant protein vaccines, which can be used without restriction in vaccine-booster programs.

**Key points:**

*• ChAdOx1 adenovirus vaccine (AZD1222) may not be effective in boosting anti-SARS-CoV-2 immunity*

*• A recombinant RBD protein vaccine is effective in boosting anti-SARS-CoV-2 immunity in mice*

*• Antibodies elicited by the rRBD-delta vaccine persisted for up to 3 months in mice*

**Supplementary Information:**

The online version contains supplementary material available at 10.1007/s00253-023-12927-0.

## Introduction

Despite global efforts to protect the population against SARS-CoV-2 through vaccination, the virus has continued to evolve and the emergence of new variants has threatened human health and the economy for the past 4 years (https://www.who.int/activities/tracking-SARS-CoV-2-variants). In this transition to the endemic phase, COVID-19 vaccination will likely become seasonal, based on antigens that will be updated as required (Zarębska-Michaluk et al. 2022; https://www.fda.gov/news-events/press-announcements/fda-takes-action-updated-mrna-covid-19-vaccines-better-protect-against-currently-circulating). Adding to this challenge is the decline in the protective capacity of available vaccines a few months after their first application (Lopez Bernal et al. 2021a; Shrotri et al. 2021), which has led the World Health Organization (WHO) to recommend booster doses of approved vaccines, several of which are still formulated with the original Wuhan virus variant. This has resulted in an increased demand for vaccines and the need to develop new, more effective vaccines that include new circulating SARS-CoV-2 variants, even in countries with high vaccination coverage.

Several vaccines currently applied in various countries use nonreplicating vectors to deliver either the full-length SARS-CoV-2 spike (S) protein or its receptor-binding domain (RBD) (Chavda et al. 2023). For instance, the AZD1222 vaccine uses the chimpanzee adenovirus vector (ChAdOx-1) (Mallapaty and Callaway 2021), whereas the single-dose Johnson & Johnson/Janssen vaccine (Ad26.COV2.S) expresses the full-length S protein in modified human adenovirus 26 (Ad26) (Sadoff et al. 2021); CanSino Biologics (Ad5-nCoV) uses nonreplicating human adenovirus 5 (Ad5) as an expression platform for protein S (Zhu et al. 2021), and Sputnik V, developed by the Gamaleya Research Institute of Epidemiology and Microbiology in Russia, uses a first dose of the S protein expressed in modified Ad5 human adenovirus and a second dose in the Ad26 adenovirus (Zhang et al. 2021). These vaccines induce protection by eliciting both humoral and cellular immune responses against all immunogenic components of the formulated vaccine (Sadarangani et al. 2021). This strategy is also being used to develop an intranasal vaccine against COVID-19 (Jung et al. 2023). Thus, immunization with adenovirus-vectored COVID-19 vaccines will promote immunity against the S protein (including the RBD region) but also against the adenovirus itself. Therefore, adenovirus-induced immunity could prevent the entry of adenovirus-vectored vaccines into the cell, hindering immunization (Rollier et al. 2021) and reducing vaccine efficacy. On the other hand, the relative weight of humoral and cell-mediated immunity in conferring protection against infection varies with each infectious vector. While various studies point to the critical role of the B and T cell-mediated immunity in COVID-19 infection (Le Bert et al. 2020; Melenotte et al. 2020; Nguyen-Contant et al. 2020; Laidlaw and Ellebedy 2022), the precise mechanisms underlying the differences observed in the duration of T and B cell responses induced by different vaccines are not well understood yet. On the other hand, the emergence of new variants of concern (VOCs) of SARS-CoV-2 such as delta (Jhun et al. 2021) and omicron (Gobeil et al. 2022) calls into question the efficacy of many widely used vaccines to elicit and maintain robust cross‐neutralizing antibody and T cell responses against known and new VOCs.

To address some of these pressing challenges, herein, we report the efficiency of boosters using a recombinant RBD (rRBD)-based subunit vaccine (delta variant) being developed in our laboratory, compared to that of the AZD1222 vaccine in mice. rRBD-based vaccines formulated with aluminum hydroxide as an adjuvant (including Soberana-01, Soberana Plus, and Abdala) have been reported to induce protective immune responses against SARS-CoV-2 (Aguilar-Guerra et al. 2021; Hernández-Bernal et al. 2022). These vaccines have been already administered to millions of persons with minimal side effects.

This study was aimed at comparing the efficacy of a virus-vectored vaccine to that of a recombinant protein vaccine for use in vaccine-booster programs.

## Material and methods

### Mice

Considering the minimal sex differences previously reported (Cervantes-Torres et al. 2022), 6-week-old female C57BL/6 mice were used in all experiments. The animals were purchased from Janvier Labs (France) and were maintained in the animal facilities of the Universidad Nacional Autónoma de México. All procedures were performed in accordance with national (Norma Oficial Mexicana, NOM-062-ZOO-1999) and institutional standards for the use and care of laboratory animals, and all protocols were approved by the institutional committee for the use and care of laboratory animals (permit numbers 7345, 6343).

### rRBD (delta variant) expression

The rRBD region of the S protein was recombinantly expressed to compare its immunogenicity as a booster vaccine with that of the AZD1222 vaccine, based on the chimpanzee adenovirus ChAdOx. The sequence of RBD-delta, the prevalent virus variant in Mexico at the start of this study, was used. The expression and purification of rRBD (delta variant, B.1.617.2 isolate) was described elsewhere (Camacho-Sandoval et al. 2021). Briefly, a plasmid containing the sequence of Wuhan SARS-CoV-2 RBD linked to a C-terminal histidine tag (His-Tag) was mutated to encode RBD-delta. Plasmid DNA was expanded in the *E. coli* DH5α strain, purified using a EndoFree® Plasmid Maxi Kit (QIAGEN), and used to transfect HEK 293 T cells (ATCC CRL-3216). Transfected cells were incubated for 4 days at 37 °C under 5% CO_2_, and RBD-delta was purified from culture supernatants by immobilized metal affinity chromatography (IMAC) using 5-mL His Trap™ nickel columns (GE Healthcare).

The His-tag was removed by digesting 1 mg of purified RBD-delta with tobacco etch virus (TEV) protease (New England BioLabs), following the manufacturer’s recommended protocol. Undigested His-tag-RBD, digested His-tag, and TEV (which has a His-tag by itself) were removed on nickel spin columns (New England BioLabs). The integrity and purity of the purified RBD were assessed by SEC-HPLC and SDS-PAGE, as described in Camacho-Sandoval et al. (2021). Endotoxin content was assessed with the LAL Endosafe® Kit (Charles River), following the manufacturer’s instructions.

**Fig. 1:**
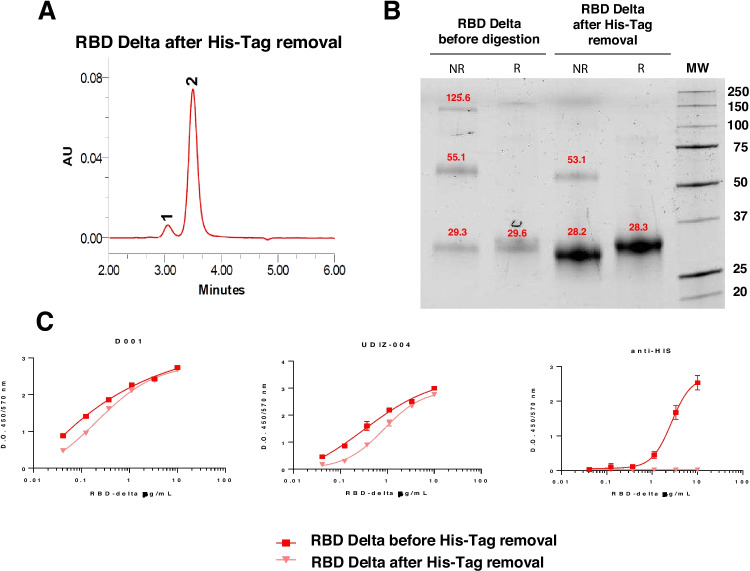
Characterization of purified and TEV protease-digested RBD-delta protein for use as an immunogen. **A** Analytical SEC-HPLC of RBD after TEV protease digestion and His-tag removal. **B** SDS-PAGE of RBD-delta before and after TEV protease digestion. The SEC profile shows two peaks, a major peak containing ~ 93% of the total protein mass and a minor peak with the remaining 7%. The major peak corresponds to monomeric RBD (~ 30 kDa). The second peak seems to be an RBD dimer, according to SDS-PAGE. **C** RBD binding, before and after His-tag removal, to two anti-RBD antibodies (D001 and UDIZ-004) and to an anti-histidine antibody (anti-His). Similar binding profiles of RBD to anti-RBD antibodies before and after digestion indicate that the protein is correctly folded after His-tag removal. RBD binding to anti-His antibody before digestion but not after His-tag removal indicates that the His-tag was successfully removed

Antibody recognition of purified rRBD-delta before and after TEV digestion was assessed by ELISA, using a commercially available anti-RBD D001 antibody (Sino Biological) and a human neutralizing antibody isolated in our laboratory (manuscript in preparation). The content of His-tag-RBD before TEV digestion and residual His-tag-RBD after digestion was assessed with an anti-His-tag antibody (Alpha Diagnostics). All assays were performed in ELISA plates (Thermo Scientific) coated with 1 µg/mL of RBD-delta before and after TEV digestion in carbonate buffer overnight at 4 °C. The plates were washed with PBS and blocked with 3% MPBS for 1 h, at room temperature. Serial dilutions of the primary antibodies in 1% MPBS were added to the coated wells and incubated for 1.5 h, at room temperature. The reaction was visualized with either 1:15,000 anti-IgG human-HRP (Abcam) or 1:20,000 anti-His-tag-HRP (Alpha Diagnostics) (Fig. 1).

### Immunization

**Fig. 2:**
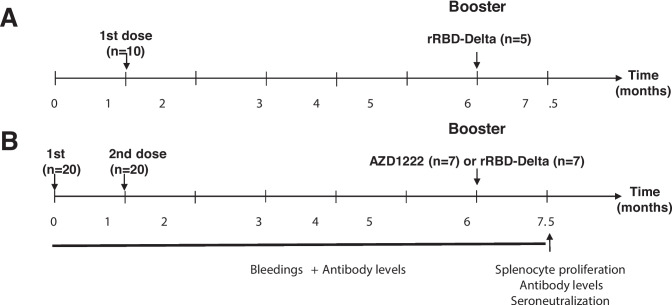
Immunization schedule to compare AZD1222 and rRBD-delta immunogenicity. Female C57Bl/6 J mice received one (**A**) or two (**B**) doses of AZD1222 30 days apart. All mice were bled monthly for 6 months to measure antibody levels. Five months after the last immunization, mice received a booster with either rRBD-delta or AZD1222, and were bled and sacrificed 45 days later to evaluate humoral and cellular responses

Two groups of 10 or 20 mice received either one (Fig. 2A) or two (Fig. 2B) doses of AZD1222 vaccine, respectively. Each mouse was administered 4.0 × 10^9^ viral particles by intramuscular (i.m.) injection into the dorsal flanks (20 µL per side) using a 27-G needle, at weeks 0 and 4 (Fig. 2). Six months later, the mice were divided into five subgroups from five to seven mice per group; one subgroup received a booster with the homologous AZD1222 vaccine by the i.m. route. The other subgroup received a subcutaneous (s.c.) booster with the rRBD-delta-based subunit vaccine (25 µg per mouse) formulated with aluminum hydroxide (0.5 mg Al^3+^) (CRODA, Denmark) to a final volume of 200 µL in the dorsal flank (27 G × 13 mm needle). The s.c. route was used to administer the rRBD-delta subunit vaccine because the volume required to inoculate 25 µg/mouse exceeded which can be inoculated intramuscularly according to ethical guidelines.

Additionally, a group of ten mice were s.c. administered with 25 µg of rRBD-delta vaccine formulated with aluminum hydroxide, as described above.

Mice were allowed food and water ad libitum and monitored daily. Blood samples were taken before immunization, every month, and 45 days after the last booster, to evaluate the humoral and cellular response of individual mice (Fig. 2). Serum samples were obtained and stored at − 20 °C until used.

### Antibody detection by ELISA

To compare the antibody response elicited by both vaccines, serum antibody levels were measured by indirect ELISA, using either Wuhan rRBD (Ayón-Núñez et al. 2022) or delta rRBD as antigens, following a previously described procedure with minor modifications (Camacho-Sandoval et al. 2021). Wells were coated with 50 µL of each rRBD (1 µg/mL) in carbonate buffer and incubated overnight at 4 °C. The plates were then washed and blocked with 150 µL of PBS-1% BSA and incubated with 50 µL of each serum diluted 1:100 in PBS-1% BSA for 1 h, at 37 °C. Antibodies were detected with 50 µL of goat anti-mouse IgG (H + L) alkaline-phosphatase conjugate (Sigma), diluted to the optimal concentration (1:2500), and followed by substrate (p-nitrophenyl phosphate, Sigma, 1 mg/mL). The reaction was stopped using 50 µL/well of 2N NaOH, and absorbance values were determined at 405 nm in a microplate spectrophotometer reader (Sinergy, Biotek, USA).

Serum levels of antibodies against the adenovirus vector were determined by indirect ELISA, using the AZD1222 vaccine as antigen. Wells were coated with 50 µL of each rRBD (0.5 µg/mL) in carbonate buffer and incubated overnight at 4 °C. The plates were then washed and blocked with 150 µL of PBS-1% BSA and incubated with 50 µL of each serum diluted 1:50 in PBS-1% BSA for 1 h, at 37 °C. Antibodies were detected with 50 µL of goat anti-mouse IgG (H + L) alkaline-phosphatase conjugate (Sigma), diluted to the optimal concentration (1:2500), and followed by substrate (p-nitrophenyl phosphate, Sigma, 1 mg/mL). The reaction was stopped using 50 µL/well of 2N NaOH, and absorbance values were determined at 405 nm in a microplate spectrophotometer reader (Sinergy).

### Adenovirus-specific response detected by Western blot

To detect the presence of anti-adenovirus antibodies previously elicited by vaccination, 3 × 10^9^ adenoviral vector particles in the AZD1222 vaccine were resolved by 12% SDS-PAGE and then transferred to a PVDF membrane to determine immune recognition by Western blot. The membranes were blocked with 3% BSA in PBS-Tween (PBS-T) for 2 h and incubated with sera from non-immunized mice or mice immunized with one or two doses of the AZD1222 vaccine. Then, the membranes were washed with PBS-T and incubated for 1 h with secondary HRP-conjugated anti-mouse IgG polyclonal antibody (Invitrogen) 1:2500. The reaction was visualized using 3 mg/mL of 3,3-diaminobenzidine (Sigma) in PBS-T and 30% hydrogen peroxide.

### Cellular immune response

To compare the cellular immune response elicited by both vaccines, splenocytes were obtained from immunized and control (non-immunized) mice. A total of 0.5 × 10^6^ cells per well were cultured in 96-well plates. To evaluate the antigen-specific T cell response, the cells were first incubated with cell trace violet (CTV) (Invitrogen) following the manufacturer’s recommended protocol. The cells were cultivated at 37 °C under 5% CO_2_ in the presence of anti-CD28 (1 µg/mL) (TONBO Biosciences), either alone (not stimulated), with rRBD-delta (20 µg/mL), or PepMix™ SARS-CoV-2 (S-RBD B.1.617.2, 1 µg/mL) (JPT). Splenocytes stimulated with anti-CD3 plus anti-CD28 (1 µg/mL each) (TONBO Biosciences) were used as a positive control, and cells cultured with CD28 only were used as a negative control. After 3 days of culture, the cells were re-stimulated with PMA (40 ng/mL) and Ionomycin (400 ng/mL) in the presence of the protein transport inhibitor GolgiPlug (Becton Dickinson) for 5 h. The cells were then stained for 20 min at room temperature with anti-CD4 APC (Biolegend), anti-CD8 PE (TONBO Biosciences), and Zombie Nir (Biolegend) as a viability dye. Then, the cells were permeabilized with Fix/Perm buffer (TONBO Biosciences) for 1 h at room temperature, and Fc receptors were blocked with anti-CD16/32 (Biolegend) for 20 min at 4 °C, followed by intracellular staining with anti-IFNγ BV510 (Biolegend), for 30 min at 4 °C. Samples were acquired in an Attune NxT acoustic focusing flow cytometer (Thermo Scientific) and analyzed with the software Flow Jo v.10.8.1 (Becton Dickinson). To determine the antigen-specific response, the percentage of CD4+ and CD8+ IFNγ+ proliferating T cells was calculated after subtracting the value of unstimulated controls in all conditions.

### Microneutralization assays

To compare the capacity of antibodies elicited by both vaccines to prevent the entry of virus into the cells, microneutralization (MN) assays were performed on twofold serial dilutions of serum samples from control and vaccinated mice in Eagle’s MEM added to an equal volume (50 µL) of SARS-CoV-2 virus at an MOI of 0.1. The virus used corresponds to a sequence showing 100% identity with the USA/CO-CDPHE-2100177494/2020 (GenBank ON228044.1) (Ayón-Núñez et al. 2022). The antibody/virus mixture was incubated at 37 °C for 1 h and then added to VERO E6 cell monolayers for 3 days at 37 °C under 5% CO_2_. The resulting cytopathic effect (CPE) was observed every day under a microscope. The cells were washed with PBS and fixed with ethanol to acetone (1:1) for 15 min and stained with violet crystal for 20 min. Positive (with a pre-screened lytic capacity) and negative (no virus added) controls were included in the assay. Antibody titers were expressed as the log_2_ maximum dilution at which the serum inhibited CPE. This procedure was performed in a BSL3 laboratory.

### Clinical and pathological evaluation in vaccinated and non-vaccinated mice

To assess the safety of the rRBD-delta vaccine, the number of deaths or apparent undesirable clinical signs was registered. The body weight of immunized and non-immunized mice was measured throughout the study period. At the end of the experiment, the mice were humanly sacrificed under anesthesia. The heart, lung, thymus, kidney, urinary bladder, liver, small intestine, stomach, colon, cerebrum, cerebellum, adrenal glands, mesenteric lymph node, pancreas, inoculation site, uterus, ovaries, and testes were fixed in 10% formaldehyde and embedded in paraffin. Serial sections, 3 µm thick, were prepared from non-consecutive areas and stained with hematoxylin and eosin (H&E). At least two sections per tissue from each animal were examined for the presence of histological abnormalities (magnification, 400 × and 100 ×).

### Statistical analysis

A normality test (D’Agostino-Pearson) was performed to determine the normal distribution in our experimental groups. Data groups with normal distribution were compared by one-way ANOVA followed by either Dunn’s multiple-comparison test or unpaired t-test using the software GraphPad Prism v.5.03. Differences were considered statistically significant at *P* < 0.05.

## Results

### Expression, purification, and characterization of rRBD-delta produced in HEK293 cells

rRBD-delta was expressed, purified, and TEV-digested to compare its immunogenic capacity with that of the AZD1222 vaccine. Figure 1A shows the analytical SEC-HPLC of RBD-delta after the removal of the His-tag by digestion with TEV. The SEC profile showed two peaks, a large peak containing ~ 93% of the total protein and a minor peak with the remaining 7%. The large peak corresponded to the monomeric form of RBD-delta (~ 30 kDa), whereas the second one seemed to consist of dimers, judging by its apparent mobility on SDS-PAGE. This interpretation would be supported by the SDS-PAGE analysis of rRBD-delta before and after digestion with TEV (Fig. 1B). Two bands were observed under non-reducing conditions, in contrast to the single band (~ 30 kDa) found under reducing conditions. The molecular mass of RBD before and after digestion differed by ~ 1 kDa, demonstrating His-tag removal.

The profile of recognition of rRBD-delta before and after His-tag removal by anti-RBD (D001 and UDIZ-004) and anti-His antibodies is shown in Fig. 1C. The patterns of antibody recognition indicate that rRBD-delta was properly folded after His-tag removal. Moreover, rRBD-delta recognition by anti-His antibodies before digestion but not after His-tag removal demonstrated again that the tag was successfully removed. It should be noted that rRBD-delta was produced and formulated with aluminum hydroxide as an adjuvant under good manufacturing production (cGMP) conditions.

### Levels of anti-RBD antibodies in mice vaccinated with one or two AZD1222 doses

**Fig. 3:**
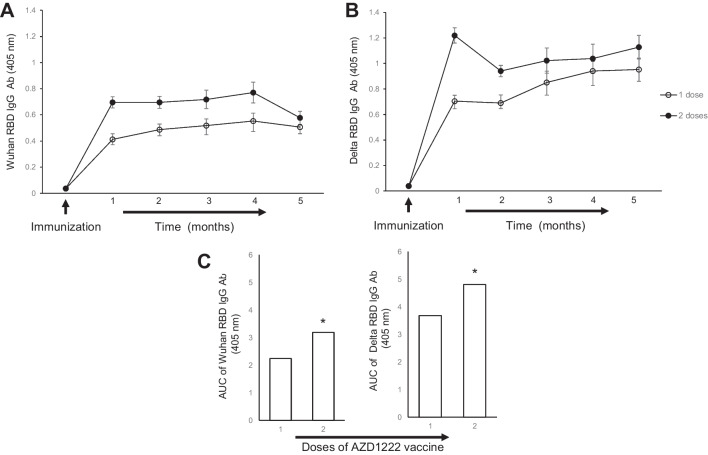
Humoral response induced by the AZD1222 vaccine. Antibody levels induced by one or two AZD1222 doses (dose = 4 × 10^9^ viral particles per mouse in 40 µL) in female C57Bl/6 J mice (*n* = 10) were measured monthly for 5 months after the last immunization. Antibodies were measured using Wuhan (**A**) or delta (**B**) RBD antigen in ELISA. Asterisks indicate significant differences when mice received two vaccine doses (**C**). Data were analyzed by one-way ANOVA followed by Dunn’s multiple-comparison test

The levels of anti-RBD antibodies induced by one or two AZD1222 doses were measured by ELISA using the Wuhan (Fig. 3A) or delta (Fig. 3B) RBD variants, before immunization and every month for the next 5 months, to evaluate the persistence of antibody levels. As shown, antibody levels were significantly increased 30 days after immunization, and this increase was more marked with two vaccine doses. As shown in Fig. 3A, antibody levels began to decrease 5 months after a two-dose immunization with AZD1222. The overall level of antibodies during the time is shown in Fig. 3C. Significantly higher antibody levels were measured when two AZD1222 doses were administered and rRBD-delta was used as antigen source for ELISA.

### Boosting efficacy was higher for rRBD-delta than for AZD1222

#### Antibody levels

**Fig. 4:**
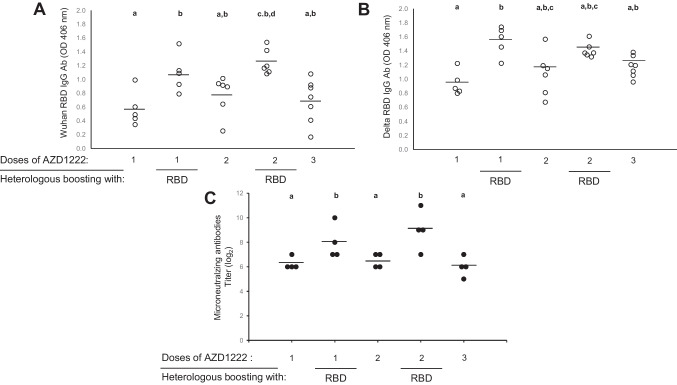
Boosting efficacy was higher with rRBD-delta than with AZD1222. Humoral immunity induced by one or two AZD1222 doses (dose = 4 × 10^9^ viral particles per mouse in 40 µL) in C57Bl/6 J female mice (*n* = 5 to 7 per group) with either an AZD1222 or rRBD-delta boost, 45 days after the last immunization. Antibody levels detected by ELISA using **A** Wuhan RBD or **B** RBD-delta. **C** Wuhan SARS-CoV-2 antibody microneutralization titers. Data were analyzed by one-way ANOVA followed by Dunn’s multiple-comparison test

The adenovirus vector-based vaccine AZD1222, which encodes SARS-CoV-2 S protein, elicited a robust antibody response against the S protein and against the adenovirus vector itself (Barnes et al. 2012; Folegatti et al 2020). Adenovirus vector-induced immunity could interfere with the efficacy of the booster. To test this possibility, considering that AZD1222-induced anti-RBD antibody levels started to decrease 5 months after the last immunization (Fig. 3A), mice were boosted with either rRBD-delta or AZD1222, and the induced immunity was compared. As shown, antibody levels (Fig. 4A and B) and neutralizing capacity (Fig. 4C) were significantly higher after a rRBD-delta booster than with AZD1222 in mice that received one or two doses of the latter.

#### Adenovirus proteins induced specific immunity after different AZD1222 doses in mice and human

**Fig. 5:**
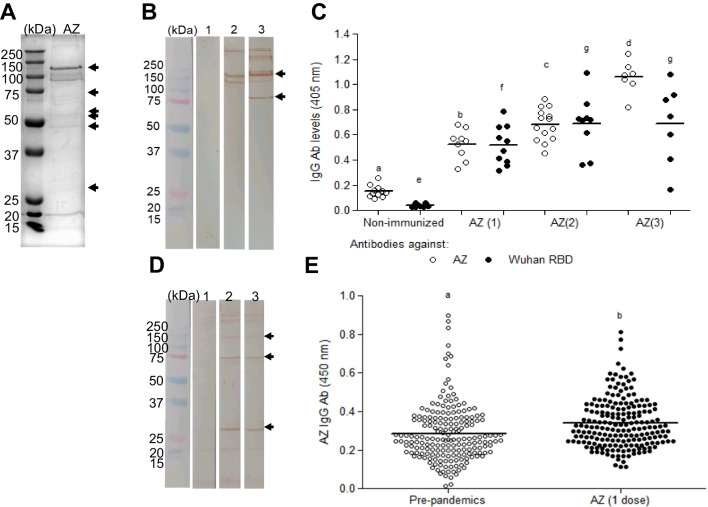
Adenovirus proteins induced specific antibodies after AZD1222 vaccination in mice and humans. **A** 3 × 10^9^ AZD1222 vaccine (AZ) viral particles were resolved by 12% SDS-PAGE. Proteins were visualized by Coomassie Blue staining. Adenovirus (Adv) proteins are labeled with arrows. **B** Adv proteins were transferred to a PVDF membrane and probed with serum samples from non-immunized mice (lane 1); mice immunized with one (lane 2) or two doses (lane 3) of AZ (4 × 10^9^ viral particles per mouse in 40 µL), washed with PBS-T, and incubated for 1 h with secondary HRP-conjugated anti-mouse IgG polyclonal antibody. The reaction was visualized using 3 mg/mL of 3,3-diaminobenzidine in PBS-T and 30% hydrogen peroxide. **C** Antibody levels detected by ELISA using AZ (○) or Wuhan RBD (●) protein. Different letters indicate significant differences between groups by two-tailed unpaired t-test (*P* < 0.05). **D** 3 × 10^9^ viral particles in AZD1222 were resolved by 12% SDS-PAGE. Proteins were transferred to a PVDF membrane to determine immune recognition by Western blot. The membranes were blocked with 3% BSA in PBS-Tween (PBS-T) for 2 h and incubated with sera from non-vaccinated human subjects (lane 1), human patients with one (lane 2) or two (lane 3) AZD1222 doses, then washed with PBS-T and incubated for 1 h with secondary HRP-conjugated anti-human IgG polyclonal antibody. The reaction was visualized using 3 mg/mL of 3,3-diaminobenzidine in PBS-T and 30% hydrogen peroxide. **E** Human serum samples collected prior to COVID-19 pandemic or from patients vaccinated with one AZD1222 dose were used to evaluate the specific response against the adenovirus vector by ELISA. *Significant differences before and after vaccination by the Mann-Whitney test (*P* < 0.05)

To detect the presence of antibodies against the viral vector, which could interfere with the virus entry into host cells, reducing S protein expression and inducing immunity, mouse and human serum samples were tested before and after immunization (Fig. 5).

Both the presence of adenovirus proteins in the AZD1222 vaccine (Fig. 5A) and anti-adenovirus antibodies were detected in mice immunized with AZD1222 (Fig. 5B and C). As shown, significantly increased levels of antibodies against adenovirus proteins were observed after the first AZD1222 dose (Fig. 5B and C). Similarly, the levels of anti-RBD antibodies were significantly increased after the first AZD1222 dose (Fig. 5C).

No anti-adenovirus antibodies were detected by Western blot in serum samples from human patients before vaccination (Fig. 5D). As expected, several adenovirus proteins were recognized after one or two vaccine doses. In line with these results, higher levels of specific anti-adenovirus antibodies were detected by ELISA in serum samples from human patients who received one AZD1222 dose than in serum samples prior to the pandemic (Fig. 5E).

#### Cellular immunity

**Fig. 6:**
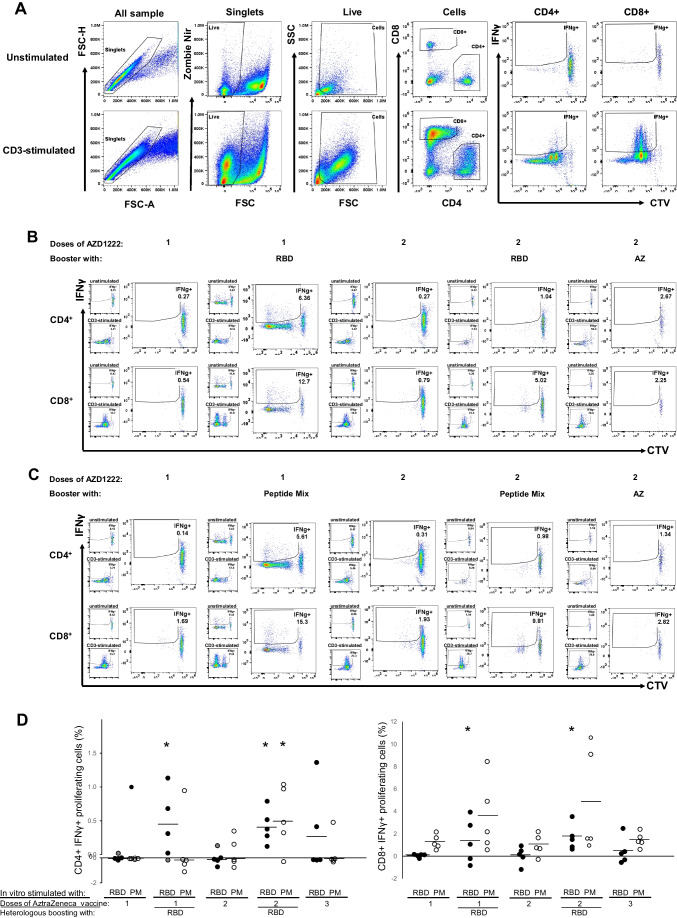
Cellular immune response in mice vaccinated with AZD1222 (AZ) and boosted with AZ or rRBD-delta. **A** Plots showing the gating strategy to analyze CD4 and CD8 T cells in nonstimulated and CD3-stimulated controls. The cells were stained with zombie NIR and fluorescent CD4, CD8, and IFNγ antibodies. A region was selected for single living cells. IFNγ+CTV low cells were selected for analysis. Splenocytes from immunized mice (*n* = 5 per group) were stained with CTV and stimulated with **B** RBD-delta or **C** a peptide mixture for 3 days. **D** Summary of proliferation analysis showing percentages of CD4+/IFNγ+ and CD8+/IFNγ+ antigen-specific cells from individual mice immunized once, twice, or three times with AZD1222 and boosted or not with RBD. Asterisks indicate statistically significant differences between immunized mice with or without boost (*P* < 0.05)

Since cellular immunity is required to ensure a long-term memory, antigen-specific response to vaccines, T cell responses specific for rRBD-delta were evaluated in mice that received one AZD1222 dose and a booster with either RBD or AZD1222. Splenocytes were stimulated with either the rRBD-delta protein or a pool of 53 peptides from RBD-delta for 72 h and analyzed by flow cytometry. The gating strategy was designed to identify proliferating, antigen-specific CD4+IFNγ+ or CD8+IFNγ+ cells. Representative plots of the above cell populations show the gating strategy used for both unstimulated and CD3-stimulated cells and evaluate the percentage of IFNγ + T cells from immunized mice (Fig. 6A). Proliferation assays showed that the percentage of CD4+IFNγ+ and CD8+IFNγ+ T cells from mice administered with one, two, or three AZD1222 doses did not increase significantly when stimulated with either rRBD (Fig. 6B) or a mixture of rRBD-delta-derived peptides (Fig. 6C) with respect to cells treated with medium alone (unstimulated). In contrast, the percentage of CD4+IFNγ+ or CD8+IFNγ+ T cells from mice vaccinated with one or two AZD1222 doses and boosted with the rRBD-delta vaccine was significantly increased when stimulated with rRBD with respect to non-boosted mice. The rRBD-delta-derived peptide mixture also induced a significant increase in the percentage of CD4+IFNγ+ cells in mice that previously received two AZD1222 doses and were subsequently boosted with rRBD-delta (Fig. 6D).

#### Persistence of antibody levels induced by the rRBD-delta vaccine

**Fig. 7:**
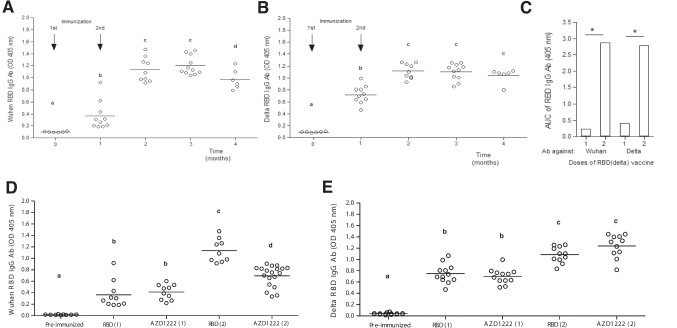
AZD1222 and rRBD-delta induced similar antibody levels. Female C57Bl/6 J mice (*n* = 10) received 25 µg of the formulated rRBD-delta vaccine. Antibody levels were detected by ELISA using either **A** Wuhan RBD or **B** RBD-delta as antigen source, 1, 2, and 3 months after the last immunization. **C** Significant differences when mice received two doses of the rRBD-delta vaccine. IgG antibody levels induced 30 days after the administration of one or two doses of AZD1222 (dose = 4 × 10^9^ viral particles per mouse in 40 µL) or rRBD-delta (25 µg per mouse) in female C57Bl/6 J mice, detected by ELISA using **D** Wuhan RBD or **E** RBD-delta as antigen source. Different letters indicate significant differences between groups, determined by one-way ANOVA followed by unpaired *t*-tests (*P* < 0.05)

High antibody levels were detected for the next 3 months after immunization with rRBD-delta (Fig. 7B). The levels of anti-Wuhan RBD antibodies were slightly decreased 4 months after immunization (Fig. 7A).

#### Similar immunity levels were induced by one or two AZD1222 or RBD-delta doses

Administering one or two doses of rRBD-delta vaccine induced similar antibody levels as the AZD1222 vaccine when detected with RBD-delta (Fig. 7B) nor Wuhan RBD in ELISA (Fig. 7A).

### Sub-chronic toxicity studies

No clinical or pathological signs were associated with rRBD-delta immunization. No deaths or apparent clinical signs were observed. The weight of the animals remained statistically unchanged before and after immunization (Figure S1). In addition, histological examination showed no abnormalities in the brain, kidney, spleen, pancreas, heart, small intestine, or colon of immunized animals (data not shown).

## Discussion

Several vaccines against SARS-CoV-2 have been developed with an unprecedented speed. In Mexico, conventional inactivated vaccines (Sinovac, Sinopharm), adenovirus (Ad)-vectored vaccines (CanSino Biological, AstraZeneca, Gamaleya, and Johnson & Johnson/Janssen), and mRNA vaccines (Pfizer and Moderna) were applied during the health emergence. Both Ad vector and mRNA vaccines promote S protein expression to induce the production of serum IgG and neutralizing antibodies (Dai and Gao 2020). Long-term studies in individuals immunized with inactivated and mRNA vaccines have demonstrated the persistence of B and T cell responses for at least 6 months after the second dose (Doria-Rose et al 2021; Goel et al. 2021; Liao et al. 2021). On the other hand, while Ad vector-based vaccines have proved to be highly immunogenic (Barnes et al. 2012; Stephenson et al. 2021), pre-existing immunity against the human adenovirus (HAd)-derived vector serotype 5, which is widespread in the human population, especially in Africa (Nwanegbo et al. 2004), limits their efficacy. Various strategies have been developed to circumvent this flaw, including the use of non-human vectors (chimpanzee viruses), rare serotypes such as Ad26, or recombinant Ads. The use of heterologous vaccines provides another means to overcome an ineffective response. Previous studies have demonstrated that a primary booster with a heterologous COVID-19 mRNA vaccine after a single Johnson & Johnson/Janssen (Ad.26.COV2.S) vaccine or a two-dose schedule with the homologous AZD1222 (ChAdOx1-S) vaccine induced an increased response of neutralizing antibodies after the heterologous booster (Liu et al. 2021; Atmar et al. 2022).

Vaccination against COVID-19 has led to a large reduction in the prevalence of severe disease, although no vaccine is able to effectively control the infection transmission (Sadarangani et al. 2021). Even though the extent of vaccination-induced protection is still uncertain, health authorities in Western countries have approved the application of booster doses to ensure that high immunity levels against the VOCs are maintained in the population (Levine-Tiefenbrun et al. 2021; Joshi et al. 2021). Despite the serious ethical concerns raised by this decision when one-third of the world’s population still lacks access to any vaccine, third and even fourth vaccine doses have been administered in several countries (Barry et al. 2021; Calderon-Margalit et al. 2021). There is still limited information about which vaccines can be combined in a booster schedule to improve immunity (Lopez Bernal et al. 2021b). Given this lack of information, vaccines have been combined in several countries based on their availability rather than on evidence on the efficacy of possible combinations (Zhang et al. 2012; Burckhardt et al. 2022). In this study, we explored the induced immunity after a booster with the ChAdOx1 Ad-vectored vaccine AZD1222 and with a recombinant RBD-delta-based vaccine. A second AZD1222 dose significantly increased antibody levels (Fig. 3), as it had been demonstrated in a single-blinded phase 1/2 randomized controlled trial (Barrett et al. 2021). However, our results clearly showed the low ability of a third AZD1222 dose to increase the immune response in mice, in contrast to a third dose using a recombinant protein vaccine (Fig. 4). This result was not entirely unexpected, considering that the pre-existing immune response induced by the Ad vector can prevent the viral vector to infect cells, thus reducing the expression of the viral S protein to reinforce the immune response (Barnes et al. 2012; Shirley et al. 2020). In addition, the presence of previously induced anti-Ad antibodies could be linked to adverse effects such as Guillain-Barré syndrome, reported in individuals vaccinated with AZD1222 and Ad26.COV2. (Rzymski 2023).

The low efficacy of a third homologous dose of AZD1222 in mice was observed both in the levels of induced antibodies and their neutralization titers (Fig. 4) and in the cell-mediated immunity elicited (Fig. 5). Booster-elicited antibody levels were measured with a self-developed ELISA (Ayón-Núñez et al. 2022) based on the use of Wuhan RBD or rRBD-delta. The use of rRBD as an antigen source in ELISA allowed us to more accurately estimate the level of antibodies that can block the interaction with the ACE2 receptor and ultimately prevent cell infection. RBD proteins corresponding to the Wuhan and delta sequences were used to evaluate the induced response, considering that the AZD1222 vaccine includes the original strain, while the rRBD-based vaccine includes the delta variant. Specific antibody levels increased significantly after a boost with rRBD-delta, but not with AZD1222 (Fig. 4). Quite unexpectedly, higher levels of anti-rRBD-delta antibodies were induced with AZD1222, a result that is not consistent with evidence in human populations (Planas et al. 2021). It is feasible that RBD-delta protein could be more antigenic for a more effective recognition of the antibodies induced.

With respect to the cellular response, we evaluated the percentage of CD4+ and CD8+ cells that specifically proliferated in response to either RBD or a pool of 53 peptides (15-mers with an 11-aa overlap) derived from SARS-CoV-2 S protein RBD (Swiss-Prot ID P0DTC2; region 319–541). Only proliferating T cells expressing IFNγ were measured. As shown in Fig. 6C, a stronger cellular response was observed in cells stimulated with the peptide pool with respect to RBD-stimulated cultures. A possible explanation is that antigen presentation of the peptides in the mixture by APCs is more efficient, as they are already processed. Interestingly, the percentage of CD4+INFγ+ and CD8+IFNγ+ cells was higher when mice were boosted with rRBD-delta than when they received AZD1222 as a booster. This could be related to the induction of Ad-specific Tregs following immunocomplex uptake by dendritic cells (DCs), which promotes a tolerogenic DC phenotype (Tran et al. 2018).

To evaluate the possibility that antibodies against the chimpanzee Ad vector in AZD1222 could interfere with its entry into the cell and reduce its immunogenicity, anti-Ad antibody levels were measured using the vaccine as antigen. As shown in Fig. 7A, anti-Ad antibodies were detected by Western blot after mouse immunization. Antibody levels rose as the number of immunizations increased (Fig. 7B). To explore the relevance of these findings in humans, the presence of anti-Ad antibodies was also evaluated by Western blot (Fig. 7C) and ELISA (Fig. 7D) in human serum samples. Increased specific anti-Ad antibody levels were detected in serum samples from individuals who received a single AZD1222 dose with respect to pre-pandemic individuals (Fig. 7E).

These results point to the advantage of combining a viral-vectored vaccine with a differently designed booster, such as those based on recombinant proteins. This information does not undermine the value of viral vector vaccines. These vaccines are suitable platforms to develop new vaccines and to address local or global epidemic outbreaks with the required alacrity. However, their recurrent use should be controlled, given the counterproductive effect of the induced immunity against the vector. Among the possible vaccines to be used in long-term campaigns, those based on recombinant proteins offer several advantages. On one hand, they induce specific immunity against the pathogen protein. On the other hand, the wide experience acquired in the use of this type of vaccines may contribute to their acceptance by the open population, which, along with their ability to maintain immunogenicity in conventional cold networks, could favor their distribution and application. Conflicting results have been reported when studying anti-Ad26 neutralizing antibody responses over a 14-month period. One study concluded that anti-Ad26 antibodies do not compromise the induction of neutralizing anti-SARS-COV-2 antibodies after a boost with Gam-COVID-Vac (Byazrova et al. 2022). However, these data were obtained using a Gam-COVID-Vac vaccine based on recombinant human nonreplicating adenovirus type 26 (rAd26), which is different from the one used herein. Considering the relevance of this information, further experiments are required.

Another result that warrants specific comment is the similar level of immunity induced by two doses of rRBD and two doses of AZD1222 (Fig. 7D and E), which suggests that the ability to induce protective immunity by both vaccines is similar.

The relevance of these findings on protection could be confirmed by viral challenge experiments, which could not be performed in this study.

In conclusion, our results highlight the need to avoid or limit the use of the same viral vector when repeated immunizations are required.

## Supplementary Information

Below is the link to the electronic supplementary material.

Supplementary file1 (PDF 173 KB)

## Data Availability

The data generated and analyzed in the present study is included in this published article.
