# Supplementary material for: Caveats of chimpanzee ChAdOx1 adenovirus-vectored vaccines to boost anti-SARS-CoV-2 protective immunity in mice
Source: Appl Microbiol Biotechnol. 2024 Jan 27;108(1):179. doi: 10.1007/s00253-023-12927-0 (PMC10821985; doi:10.1007/s00253-023-12927-0)
Supplement: Supplementary file 1 — Supplementary file1 (PDF 173 KB) [file 253_2023_12927_MOESM1_ESM.pdf]

Supplemental Material

Caveats of chimpanzee ChAdOx1 adenovirus-vectored vaccines to boost anti-SARS-CoV-2 protective immunity in mice

Journal: Applied Microbiology and Biotechnology

Authors:

Jacquelynne Cervantes-Torres[a,b], Carlos Cabello-Gutiérrez[c], Dolores-Adriana Ayón-Núñez[a], Gloria Soldevila[b,d]., Roxana Olguin-Alor[b,d], Georgina Diaz[b], Gonzalo Acero[b], René Segura-Velázquez[a], Leonor Huerta[b], Isabel Gracia-Mora[e], Laura Cobos[a]., Mayra Pérez-Tapia[f], Juan C. Almagro[f], Francisco Suárez-Güemes[a], Raúl J. Bobes[b], Gladis Fragoso[2], Edda Sciutto[b]*, and Juan P. Laclette[b]*

Affiliations:

[a]School of Veterinary Medicine and [b]Biomedical Research Institute, Universidad Nacional Autónoma de México, 04510 Coyoacán, Ciudad de México, México.

[c]Instituto Nacional de Enfermedades Respiratorias "Ismael Cosío Villegas". Calzada de Tlalpan 4502, Belisario Domínguez Secc. 16, Tlalpan, 14080 Ciudad de México, CDMX.

[d]Laboratorio Nacional de Citometría de Flujo, Instituto de Investigaciones Biomédicas, Universidad Nacional Autónoma de México, 04510 Coyoacán, Ciudad de México, México.

[e] Unidad de Experimentación Preclínica. Facultad de Química. Universidad Nacional Autónoma de México, 04510 Coyoacán, Ciudad de México, México.

[f]Unidad de Desarrollo e Investigación en Bioterapeúticos (UDIBI), Escuela Nacional de Ciencias Biológicas, Instituto Politécnico Nacional, México City 11340, México.

Corresponding Author:

Edda Sciutto, Instituto de Investigaciones Biomédicas, Universidad Nacional Autónoma de México, 04510 Coyoacán, Ciudad de México, México, 52(55) 5622-3162; e-mail: edda@unam.mx

Juan Pedro Laclette, Instituto de Investigaciones Biomédicas, Universidad Nacional Autónoma de México, 04510 Coyoacán, Ciudad de México, México, 52(55) 5622-3153; e-mail: laclette@iibiomedicas.unam.mx

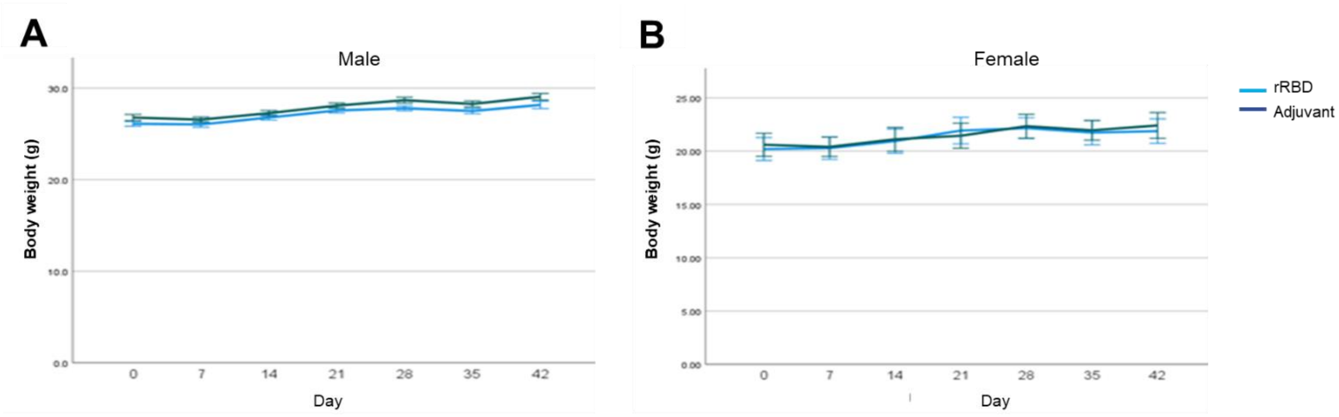

Fig. S1. Effect of rRBD-delta vaccine on body weight of mice for a 42-day period. Groups of six female C57Bl/6J mice received two doses of rRBD-delta vaccine (25 µg per mouse) (light blue lines) or aluminum hydroxide (dark blue lines), and weight was recorded every week (days 0, 7, 14, 21, 28, 35, and 42). Bars indicate the mean ± SE change in body weight of mice.
